# Global gradients in species richness of marine plankton functional groups

**DOI:** 10.1093/plankt/fbad044

**Published:** 2023-10-12

**Authors:** Fabio Benedetti, Nicolas Gruber, Meike Vogt

**Affiliations:** Environmental Physics, Institute of Biogeochemistry and Pollutant Dynamics, ETH Zürich, 8092 Zürich, Switzerland; Environmental Physics, Institute of Biogeochemistry and Pollutant Dynamics, ETH Zürich, 8092 Zürich, Switzerland; Environmental Physics, Institute of Biogeochemistry and Pollutant Dynamics, ETH Zürich, 8092 Zürich, Switzerland

**Keywords:** plankton functional groups, species diversity, global ocean, species distribution models, biodiversity–ecosystem functioning

## Abstract

The patterns of species diversity of plankton functional groups (PFGs) remain poorly understood although they matter greatly for marine ecosystem functioning. Here, we use an ensemble of empirical species distribution models for 845 plankton species to estimate the global species richness of three phytoplankton and 11 zooplankton functional groups as a function of objectively selected environmental predictors. The annual mean species richness of all PFGs decreases from the low to the high latitudes, but the steepness and the shape of this decrease vary significantly across PFGs. Pteropods, small copepods (Oithonids and Poecilostomatoids) and Salps have the steepest latitudinal gradients, whereas Amphipods and the three phytoplankton groups have the weakest ones. Temperature, irradiance and nutrient concentration are the first-order control on the latitudinal richness patterns, whilst the environmental conditions associated to upwelling systems, boundary currents and oxygen minimum zones modulate the position of the peaks and troughs in richness. The species richness of all PFGs increases with net primary production but decreases with particles size and the efficiency of the biological carbon pump. Our study puts forward emergent biodiversity–ecosystem functioning relationships and hypotheses about their underlying drivers for future field-based and modelling research.

## INTRODUCTION

Countless micro- and macroplankton drive biogeochemical cycles and ecosystem processes in the oceans (Falkowski and Oliver, 2007; Steinberg and Landry, 2017). Phytoplankton (i.e. the photoautotrophic microalgae and bacteria) carry out nearly half of the planetary net primary production (NPP; Field *et al.*, 1998), thereby fuelling the transfer of energy from the basis of all marine ecosystems to higher trophic levels. By doing so, phytoplankton control the concentration and distribution of nutrients (Sarmiento and Gruber, 2006; Martiny *et al.*, 2013; Inomura *et al.*, 2022). Phytoplankton represent a major food source for the zooplankton (i.e. the heterotrophic protists and animals) that ensure secondary production and sustain global fisheries (Beaugrand *et al.*, 2010; Steinberg and Landry, 2017). Together, phytoplankton and zooplankton modulate the biogeochemical carbon pump that helps regulate Earth’s climate by sequestrating atmospheric CO_2_ (Sarmiento and Gruber, 2006). These major ecosystem functions are ensured by more than 100 000 different species altogether, some of which remain unknown in spite of centuries of research (de Vargas *et al.*, 2015; Abreu *et al.*, 2022).

Whilst many marine plankton species remain undescribed, it is well understood that variations in evolutionary histories lead to major inter-group and inter-species differences in functional traits and thus different contributions to ecosystem functions (Litchman *et al.*, 2009, 2013; Henson *et al.*, 2019; Martini *et al.*, 2021). Major differences in cell size (or body size for multicellular organisms), morphology, elemental composition, feeding modes, life cycles or behaviours exist across and within the extant taxonomic groups of the plankton (Beaugrand *et al.*, 2010; Assmy *et al.*, 2013; Henschke *et al.*, 2016; Brun *et al.*, 2017; McConville *et al.*, 2017; Conley *et al.*, 2018; Tréguer *et al.*, 2018). For instance, diatoms take up silicic acid to build silicified cell walls that protect them from grazers and increase their sinking rates (Tréguer *et al.*, 2018), a trait that is unique in the phytoplankton. In spite of this group-specific, unifying trait, diatom species exhibit a variety of cell size, morphologies, silica content and life strategies that affect diatom population dynamics, nutrients uptake rates and carbon export fluxes (Assmy *et al.*, 2013; Leblanc *et al.*, 2018; Tréguer *et al.*, 2018). In the zooplankton, copepods alone include species of body lengths varying from 0.1 to 10 mm, with passive or active feeding techniques that modulate their grazing and mortality rates (Benedetti *et al.*, 2016; Brun *et al.*, 2017; Van Someren Gréve *et al.*, 2017). Phytoplankton-grazing crustacean zooplankton is made up of relatively more carbon than carnivorous and flux-feeding gelatinous clades such as jellyfish and tunicates (Kiørboe, 2013; McConville *et al.*, 2017; Conley *et al.*, 2018). Hence, inter- and intra-group species diversity and biotic interactions matter for marine ecosystem functioning on local to global scales (Strong *et al.*, 2015; Guidi *et al.*, 2016; Gonzalez *et al.*, 2020). However, the patterns of plankton species diversity and their underlying drivers remain poorly understood, despite more than 60 years of research (Hutchinson, 1957).

To deal with such biological complexity, functional types emerged as a concept to aggregate a large number of plankton taxonomic units (e.g. species) into fewer manageable compartments based on ecological and biogeochemical function to represent the role of plankton in mechanistic ecosystem models (Le Quéré *et al.*, 2005; Hood *et al.*, 2006). However, most of these mechanistic models are not designed to capture the vast diversity of species and traits that exists within functional types (Anderson, 2005; Le Quéré *et al.*, 2005). In addition, they are not designed to study the processes that generate plankton species diversity patterns, except for a few (Barton *et al.*, 2010; Ward *et al.*, 2012; Dutkiewicz *et al.*, 2020). Field observations remain essential to document the diversity patterns of plankton functional types, so we can unravel their abiotic drivers and then improve and benchmark new ecosystem models that better accommodate biological diversity. Recent empirical modelling studies showed that phyto- and zooplankton display latitudinal diversity gradients that resemble the ones of other marine ectotherms, with increasing number of species from the high to the low latitudes (Ibarbalz *et al.*, 2019; Righetti *et al.*, 2019; Benedetti *et al.*, 2021), a pattern hypothesized to be driven by temperature (Tittensor *et al.*, 2010). Other observational and model-based studies reported a much weaker role of temperature in shaping phytoplankton diversity (Rodríguez-Ramos *et al.*, 2015; Busseni *et al.*, 2020; Dutkiewicz *et al.*, 2020) and emphasized the role of mixing and nutrient concentrations (Barton *et al.*, 2010; Vallina *et al.*, 2014; Mangolte *et al.*, 2022). Several observation-based studies documented global and regional latitudinal diversity gradients for the following taxonomic groups and/or functional types: Diatoms (Malviya *et al.*, 2016; Endo *et al.*, 2018; Busseni *et al.*, 2020), Coccolithophores (O’Brien *et al.*, 2016; Endo *et al.*, 2018); Dinoflagellates (Chen *et al.*, 2011; Le Bescot *et al.*, 2016), Microzooplankton (Dolan *et al.*, 2016), Copepods (Woodd-Walker *et al.*, 2002; Rombouts *et al.*, 2011; Hirai *et al.*, 2020), Euphausiids (Tittensor *et al.*, 2010), Foraminifera (Lombard *et al.*, 2011; Yasuhara *et al.*, 2020; Rufino *et al.*, 2022), Radiolaria (Boltovskoy and Correa, 2017; Biard *et al.*, 2016), Chaetognaths (Miyamoto *et al.*, 2014), Pteropods (Burridge *et al.*, 2016) and hyperiid Amphipods (Burridge *et al.*, 2017). But the broad variety of scales, sampling methodologies and numerical approaches covered by these studies make inter-group and intra-group comparisons of diversity patterns difficult and differences very hard to explain. Because some groups are inadequately sampled by traditional sampling techniques (i.e. plankton nets), their contributions to community abundance, biomass and diversity have been historically underestimated, and global estimates of species diversity and composition are still missing for many key PFGs (i.e. Dinoflagellates, Ciliates, Pteropods, Tunicates, Jellyfish, Chaetognaths or Amphipods). A major consequence of these historical disparities in sampling schemes is that we are still lacking a common and unifying framework to compare large-scale species diversity patterns between groups of the marine plankton. Such a framework would enable us to test hypotheses about the relative importance of the underlying drivers of species diversity and to investigate its link with ecosystem functions such as productivity, resource use and the efficiency of export production (Cardinale *et al.*, 2012). Through the asynchronous selection of those species that are the fittest, more diverse communities tend to display a range of species capable of exploiting the various resources available (Cardinale *et al.*, 2012). This paradigm mainly emerged from local experiments on land where relatively few species were manipulated to examine the relationship between species richness and functions such as productivity or resource use (Cardinale *et al.*, 2012). Very few studies documented biodiversity–ecosystem functioning relationships for plankton communities, and they usually reported conflicting results (Ptacnik *et al.*, 2008; Cermeño *et al.*, 2013; Vallina *et al.*, 2014; Gamfeldt *et al.*, 2015; Lehtinen *et al.*, 2017; Acevedo-Trejos *et al.*, 2018; Chen *et al.*, 2019). On top of lacking fundamental knowledge on the global diversity gradients of various plankton groups, we remain even further away from understanding how such diversity gradients relate to ecosystem functions like nutrient use efficiency, productivity and carbon export.

Here, we use the recent compilations of plankton species occurrences and ensemble species distribution models (SDMs) framework of Benedetti *et al.* (2021) to (i) hierarchize the environmental predictors of species distributions that are then used to (ii) estimate the global richness patterns for 14 different functional groups (instead of broad trophic levels) and evaluate how they covary with key ecosystem properties (i.e. NPP, carbon export efficiency or higher trophic level diversity).

## METHODS

### Plankton occurrence data

Species-level plankton occurrences (presence-only) were retrieved from two recent efforts aiming to describe and model phyto- and zooplankton communities in the global open ocean (Righetti *et al.*, 2019; Benedetti *et al.*, 2021). Plankton are commonly divided between two trophic levels: the photoautotrophic phytoplankton and the heterotrophic zooplankton. Both categories comprise a tremendous variety of species belonging to various clades and are characterized by different biological requirements and thus niche dimensions. The steps described below, from data acquisition to SDMs parameterization, reflect this major initial dichotomy.

The phytoplankton species occurrence data used here were compiled by Righetti *et al.* (2020) and stem from various sources: Global Biodiversity Information Facility (GBIF; https://www.gbif.org), Ocean Biogeographic Information System (OBIS; https://www.obis.org), Villar *et al.* (2015) and the MARine Ecosystem biomass DATa (MAREDAT) (Buitenhuis *et al.*, 2013). The dataset gathers > 10^6^ occurrences for nearly 1700 species sampled through diverse techniques within the monthly climatological mixed layer depth, at an average depth of 5.4 ± 6.9 m (mean ± SD) for the 1800–2015 period. Phytoplankton species names were corrected and harmonized following the reference list of Algaebase (http://www.algaebase.org/). This dataset has been used to derive a surface open ocean estimate of the global phytoplankton diversity that properly accounts for sampling spatial–temporal biases and that was validated against independent data (Righetti *et al.*, 2019; Benedetti *et al.*, 2021).

The zooplankton species occurrences used here were compiled by Benedetti *et al.* (2021). In short, occurrences were primarily retrieved from OBIS and GBIF and complemented by the observations from Cornils *et al.* (2018) and Bednaršek *et al.* (2012). The data cover all main extant marine zooplankton clades (Suthers *et al.*, 2009). Occurrences that are not representative of contemporary open ocean plankton communities were discarded based on the following criteria: (i) a missing spatial coordinate, (ii) incomplete sampling date (d/m/y), (iii) year of collection older than 1800, (iv) no sampling depth provided and sampling depth >500 m (i.e. excluding taxa mainly inhabiting the deep ocean whilst accounting for those performing vertical migrations), (v) not identified to the taxonomic species level, (vi) issued from drilling holes or sediment samples, (vii) associated with monthly surface salinity values < 20 and (viii) collected in a 1 × 1 grid cell displaying a seafloor shallower than 200 m (i.e. excluding coastal areas). Zooplankton species name were carefully harmonized following the reference list of the World Register of Marine Species (WoRMS; http://www.marinespecies.org). The final zooplankton dataset compiled occurrences for > 3000 different taxa spanning the main zooplankton clades and functional groups.

### Plankton functional groups

The definition of plankton functional groups (PFGs) depends on the scientific question addressed (Le Quéré *et al.*, 2005; Hood *et al.*, 2006). Most of the time, taxa are grouped into PFGs based on the performance of particular biogeochemical functions (e.g. calcification vs. silicification; Iglesias-Rodríguez *et al.*, 2002) and/or size classes that relate to their trophic level in food-webs and how they contribute to pathways of carbon fluxes (e.g. nano-, micro- or mesoplankton). Here, we focus on 14 PFGs (2 for traditional phytoplankton, 1 for mixotrophic plankton and 11 for zooplankton) displaying at least five different species whose distributions could be modelled from the available occurrences data (see section Species Distribution Modelling). The PFGs investigated and their main functions are described in Table I. These PFGs included taxa from either the same size class and/or that are known to fill the same trophic niche (i.e. they rely on similar energy sources of inorganic and organic carbon and/or nutrients and/or are eaten by similar organisms) as a result of similar feeding traits (Kiørboe, 2011). Because the observational datasets do not cover the smallest size classes of the plankton (Righetti *et al.*, 2020; Benedetti *et al.*, 2021), some key functional groups (e.g. N2-fixing prokaryotes, picophytoplankton, microzooplankton) could not be integrated in this study.

**Table I:** Overview and description of the 14 PFGs whose species richness is modelled in the present study

PFG	Number of species modelled	Size range	Main ecological and biogeochemical functions performed
Coccolithophores	26	Nanophytoplankton (2–20 μm)	Calcifying phytoplankton responsible for half of marine CaCO_3_ (calcite) fluxes and that trigger large blooms at high latitudes, thus influencing marine primary production, alkalinity, carbonate and carbon chemistry on short to geological time scales (Monteiro *et al.*, 2016).
Dinoflagellates	155	Nano- and microplankton (2–200 μm)	Mixotrophic plankton that can perform photosynthesis through diverse mechanisms but that can also be heterotrophs (“mixoplankton”; Mitra *et al.*, 2023). Forty-five point five percent of the Dinoflagellates species modelled are constitutive mixoplankton, and 14.3% are specialized non-constitutive mixoplankton. The mixoplankton type of the remaining 40% remains unknown, although some of them (*Protoperidinium* spp.) have been described as heterotrophic “protozooplankton” (Schneider *et al.*, 2020; Mitra *et al.*, 2023). Several Dinoflagellates can trigger harmful algal blooms (Gómez, 2012).
Diatoms	160	Microphytoplankton (20–200 μm)	Large silicifying phytoplankton often considered as the main contributors to phytoplankton biomass, especially in cold and nutrients-enriched waters. Diatoms deplete silicic acid concentrations and are a key contributor to carbon export through diverse processes: high sinking rates through grazing, ballasting of large and mineralized cells, resting spores, etc. (Sarthou *et al.*, 2005; Tréguer *et al.*, 2018).
Calanoids	221	Small to large mesozooplankton (0.2–20 mm)	Crustacean zooplankton considered to be the main grazer of phytoplankton through active current feeding (Kiørboe, 2011; Benedetti *et al.*, 2022). This order of copepods strongly contributes to the biological carbon pump not only through phytoplankton and microzooplankton grazing but also through the production of faecal pellets and by performing diel vertical migrations (Jónasdóttir *et al.*, 2015; Steinberg and Landry, 2017).
Oithonids	12	Small mesozooplankton (often < 1 mm)	Small copepods that feed more passively than calanoids (i.e. passive ambush feeders; Kiørboe, 2011; Benedetti *et al.*, 2022). *Oithona* spp. are omnivorous (e.g. feed on microzooplankton, small phytoplankton and detritus) and considered the most ubiquitous and abundant copepods worldwide (Gallienne and Robins, 2001). Oithonid copepods present lower rates of growth, mortality, feeding and reproduction than calanoid copepods of comparable size (Cornwell *et al.*, 2020).
Poecilostomatoids	41	Small mesozooplankton (often < 2 mm)	Small copepods that feed on smaller zooplankton (e.g. nauplii and ciliates) through visual ambush feeding (Corycaeidae; Benedetti *et al.*, 2022) or attach themselves on detritus aggregates, thus contributing to the remineralization of sinking particulate organic matter (Oncaeidae; Benedetti *et al.*, 2022). Like Oithonids, they display lower physiological rates than calanoids and thrive in oligotrophic conditions.
Jellyfish (holoplanktonic Cnidaria and Ctenophora)	69	Macrozooplankton (>20 mm)	Gelatinous zooplankton that perform passive ambush feeding to capture smaller zooplankton (Kiørboe, 2011). Larger jellyfish serve as important prey items for endangered species (e.g. turtles). They can form local outbursts of gelatinous biomass that efficiently export carbon (Lebrato *et al.*, 2019) and that may cause significant economic damages (Richardson *et al.*, 2009). As jellyfish prey on food items of several orders of magnitude smaller than them, they divert the classical 10:1 predator–prey size ratio prevailing in the oceans (Andersen *et al.*, 2016).
Euphausiids (Krill)	51	Macrozooplankton (>10 mm)	Crustacean macrozooplankton that actively graze on large phytoplankton and mesozooplankton through filter feeding. Compared to mesozooplankton, Euphausiids not only display very high swimming speeds enabling them to perform larger vertical migrations but also show higher feeding rates leading to the production of very large faecal pellets. Altogether, these traits make Euphausiids a major components of biogeochemical cycles and food-webs in regions like the Southern Ocean where they can reach tremendous biomass levels (Cavan *et al.*, 2019).
Amphipods (Hyperiids)	20	Meso- to macrozooplankton (>2 mm)	Crustacean zooplankton specialized in commensalism and parasitism of gelatinous zooplankton (Cnidaria and Tunicata; Burridge *et al.*, 2017; Havermans *et al.*, 2019). A few species (e.g. *Themisto gaudichaudii*) are free-living predators whose swarms cruise through the water column to prey on mesozooplankton. Their impact on carbon cycling and biomass transfers remain very poorly understood, although they can dominate biomass levels locally.
Chaetognaths	27	Large mesozooplankton (1–100 mm)	Exclusively marine group of translucent arrow-shaped worms that are found worldwide and yet remain poorly studied. They feed on smaller mesozooplankton (i.e. copepods) through active ambush feeding based on mechanoreception (Kiørboe, 2011).
Pteropods	19	Mesozooplankton (<20 mm)	Calcifying zooplankton that performs passive filter feeding by deploying a mucus net to aggregate the sinking particles they feed on (Kiørboe, 2011). We here focus on the Thecosomata, which constitute > 90% of extant Pteropod species (Peijnenburg *et al.*, 2020). The aragonite shells of the Thecosomata are more soluble than the calcite-made platelets of Coccolithophores and are sensible to pCO_2_ variations.
Thaliaceans (mostly Salps)	12	Meso- to macrozooplankton (0.5–200 mm)	Barrel-shaped gelatinous zooplankton with relatively complex nervous and digestive systems. Salps are colony-forming omnivorous passive filter feeders that pump water into their body where particles are retained on a mucous net. This strategy allows them to capture particles ranging from 1 μm to 1 mm whereas crustacean filter feeders (i.e. calanoids and euphausiids) usually feed on particles < 200 μm. Salps contribute to an efficient pathway of carbon export as they produce large and fast-sinking faecal pellets (Henschke *et al.*, 2016).
Appendicularians	6	Mesozooplankton (<10 mm)	Appendicularians are pelagic tunicates that perform passive filter feeding by creating a gelatinous “house,” which they use for sheltering and aggregating the particles they feed on (Kiørboe, 2011). Discarded houses of appendicularians are a great source of food for detritivorous copepods (*Oncaea* spp. and *Microsetella* spp.) and a potentially strong pathway of particles export, as such houses can reach 1 m in diameter (Alldredge, 1972; Kiørboe, 2011). Like Thaliaceans, their contribution to pathways of particles export remains too poorly studied (Alldredge, 1972; Katija *et al.*, 2017).
Foraminifera	26	Mesozooplankton (<1 mm)	Calcifying protists characterized by calcareous shells displaying chambered and perforated tests from which their ectoplasm can emerge to catch food items (i.e. passive ambush feeders). Many extant planktic Foraminifera also bear endophotosymbionts that make these organisms mixotrophic instead of heterotrophic (Schiebel and Hemleben, 2005; Kimoto, 2015; Mitra *et al.*, 2023). Together with Coccolithophores and Pteropods, they are the main producers of CaCO_3_ in the marine plankton

In total, 845 different species could be classified into one of the above PFGs and were considered for modelling. Diatoms, Dinoflagellates and Coccolithophores constitute 47%, 45% and 8% of the phytoplankton and mixotrophic (i.e. Dinoflagellates) species, respectively. Calanoids represent 44% of all zooplankton species, and all other zooplankton groups show relative contributions to total species richness of <15% (Fig. S1; Table S1).

### Species distribution modelling

#### Model types

SDMs were developed following the standard ensemble modelling approach of Benedetti *et al.* (2021), which covers a range of model types and complexity: generalized linear models (GLMs), generalized additive models (GAMs) and artificial neural networks (ANNs). To prevent the pitfalls associated with model over-fitting (Merow *et al.*, 2014), we restricted the number of environmental predictors relative to the number of presences (see below) and SDMs were tuned to fit relatively simple response curves. All SDMs were developed using the “*biomod2”* R package (Thuiller *et al.*, 2023). We refer to Benedetti *et al.* (2021) for an exhaustive description of the SDMs parametrization as well as their sensitivity to sampling biases and input data.

#### Background data

The SDMs used here are regressive algorithms that require both presence and absence data. However, true absences are extremely difficult to ascertain, especially for small mobile species evolving in a very dynamic 3D environment. Therefore, we generated background data (also frequently called “pseudo-absences”; Barbet-Massin *et al.*, 2012; Righetti *et al.*, 2023) as a surrogate for absences. Background data are drawn to inform the SDMs as to which environmental conditions were sampled by the observations but where a species is less likely to occur (Barbet-Massin *et al.*, 2012). To do so, we used the target-group approach of Phillips *et al.* (2009), which draws background data as a function of the presence data distribution and thus does not induce additional biases and avoids misclassifying unsampled regions as unsuitable habitats. This method is one of the most suited for applying regressive SDMs to sparse plankton data (Righetti *et al.*, 2023). The PFGs were used as target-groups, meaning that the background data of each species were randomly drawn from the sites (i.e. monthly 1 × 1 grid cells) displaying at least one occurrence of their corresponding PFG whilst omitting those sites where the species was found as present. In this approach, background data represent sites, and thus environmental conditions, where a sample was taken but the species of interest was not found although species from the same functional group were observed there. Four zooplankton PFGs displayed too sparse occurrences to be considered as target-groups (Amphipods, Thaliaceans, Appendicularians and Foraminifera; Fig. S2). In this case, the background data of the corresponding species were randomly drawn from the total pool of sites with zooplankton occurrences (i.e. total-background approach; Righetti *et al.*, 2023). Here, only those sites presenting at least one occurrence from five different PFGs were considered to avoid including sites where oceanographic cruises focused on a small subset of the plankton community (e.g. only diatoms or only pteropods). For each species, 10 times more background data than presences and background data were weighted inversely proportional to the presences (Barbet-Massin *et al.*, 2012). Ultimately, all background data were matched with monthly values for the predictors considered environmental predictors for the SDMs.

#### Selection of environmental predictors

A comprehensive set of monthly climatologies for 20 environmental predictors that affect the physiology and the distribution of plankton species was implemented onto the standard 1 × 1 global cell grid of the World Ocean Atlas (WOA; see Table S2 for exhaustive references). First, 11 primary predictors were retrieved: sea surface temperature (SST, °C); surface concentration of nitrate (NO_3_^−^), phosphate (PO_4_^3−^) and silicic acid (Si(OH)_4_) (μmol L^−1^); dissolved oxygen concentration at 175-m depth (O_2_, mL L^−1^); photosynthetically active radiation (PAR; μmol m^−2^ s^−1^); surface phytoplankton chlorophyll-a (Chl-a, mg m^−3^); mixed-layer depth (MLD, m); surface wind stress (Wind, m s^−1^); surface partial pressure of carbon dioxide (pCO_2_, μatm); and eddy kinetic energy (EKE, m^2^ s^−2^) as a proxy of the strength of mesoscale activity. Then, 9 secondary predictors were derived from the 11 primary ones: PAR over the MLD (MLPAR, μmol m^−2^ s^−1^); excess of NO_3_^−^ to PO_4_^3−^ relative to the Redfield ratio (N^*^ = NO_3_^−^—16*PO_4_^3−^; Gruber and Sarmiento, 1997); and excess of Si(OH)_4_ relative to NO_3_^−^ (Si^*^ = Si(OH)_4_—NO_3_^−^; Sarmiento *et al.*, 2004). Since the distribution of macronutrients concentrations, Chl-a and EKE, were highly skewed towards low values, we examined their logarithmic transformations (log(NO_3_), log(PO_4_), log(Si(OH)_4_), log(EKE) and log(Chl-a) (based on the natural log) as additional secondary predictors as these were closer to a normal distribution.

Collinearity between predictors can increase the standard errors of regression parameters, inflate their variance in regressive models, distort importance rankings and thus bias SDMs projections (Dormann *et al.*, 2013). Therefore, we investigated predictor collinearity by computing pairwise Spearman’s rank correlation coefficients (*ρ*) from each species-specific dataset. Median pairwise correlation coefficients were derived from the distribution of species-level *ρ* values and one of two predictors from a pair displaying a median |*ρ*| > 0.75 was discarded (Dormann *et al.*, 2013). Preference was given to those predictors closest to a normal distribution (according to Shapiro–Wilk tests) and least correlated to SST.

Nutrient predictors other than N^*^ and Si^*^ tend to be strongly correlated with several pairs exhibiting median |*ρ*| > 0.75. Consequently, NO_3_^−^ and PO_4_^3−^ concentrations were discarded and only log(Si(OH)_4_) was retained as a predictor representing the global gradient in nutrient availability as its median *ρ* with SST is lower (median *ρ* = −0.15) than that for NO_3_^−^ and PO_4_^3−^ (median *ρ* = −0.51 and – 0.17, respectively). Plus, Si(OH)_4_ is a limiting nutrient for shaping diatom growth and distributions. We used its log-transform since it showed a more normal distribution than Si(OH)_4_. Log(PO_4_) has a median *ρ* with N^*^ of −0.82, so N^*^ was kept. The other cluster of highly correlated predictors comprised MLD, PAR and MLPAR (median *ρ* = −0.78 and 0.84 between MLPAR and MLD and then PAR, respectively). MLPAR was discarded as the median *ρ* between MLD and PAR was −0.69, and MLD was very weakly correlated with SST (median *ρ* = −0.07). N* and O_2_ showed a median correlation coefficient very close to the selection threshold (median rho = 0.74). N* was also found to be a relatively important covariate for constraining phytoplankton species distributions based on the tests below (see section Ranking of Predictors Importance across PFGs) as well as in previous studies (Righetti *et al.*, 2019). Furthermore, N* is more likely to be tightly linked to marine phytoplankton growth and distribution than O_2_ (Martiny *et al.*, 2013; Inomura *et al.*, 2022). Therefore, we chose N* over O_2_ here. The final 10 predictors retained for Coccolithophores, Diatoms and Dinoflagellates were SST, PAR, log(Chl-a), Wind, MLD, N^*^, Si^*^, log(Si(OH)_4_)_,_ log(EKE) and pCO_2_.

For the zooplankton groups, the same criteria were used to select their subset of predictors. The predictors at the location of the observations display similar patterns in pairwise median correlations *ρ* as seen for phytoplankton, except for the macronutrients that have even stronger correlations (all pairwise median *ρ* > 0.80) and SST (all pairwise median *ρ* < −0.75 except for log(Si(OH)_4_)). Therefore, the same predictors as for Coccolithophores, Diatoms and Dinoflagellates were retained for the zooplankton PFGs, with the addition of O_2_.

#### Ranking of predictors importance across PFGs

To further narrow down the number of PFG-specific predictors to be included in the SDMs, univariate permutation tests were performed to rank the retained predictors. For every species-specific dataset, the values of one of the predictors were randomly reshuffled (i.e. randomly re-assigned to the presence-background data) and a multivariate SDM was trained and its projections was compared to those from the non-reshuffled SDM through a Pearson’s correlation coefficient. The latter was then subtracted from 1, thus returning a quantitative score varying between 0 and 1, which indicates the importance of the reshuffled covariate for the performance of the multivariate SDM and thus for constraining the modelled species’ distribution. For each species and SDM type, 30 univariate random permutation experiments were carried out, which lead to 90 scores of relative importance per predictor. Non-parametric variance analysis (Kruskal–Wallis rank sum tests) was performed to assess the variations of each predictor’s ranking across all species modelled, phyto- and zooplankton species separately and across PFGs. Dunn’s pairwise multiple comparison rank sum tests were then applied with a Bonferroni correction to identify pairs of predictors displaying significant variations in rankings.

For each PFG, the six predictors displaying the highest scores were retained to model the distribution of the species belonging to the corresponding PFG. We retained six predictors as all the species modelled present > 75 occurrences and because we aimed to achieve a presence-to-predictors ratio > 12:1, which is higher than the 10:1 ratio recommended by Guisan *et al.* (2017). Concomitantly, each SDM was run 10 times with a five-fold cross validation and the resulting True Skill Statistics (TSS; Allouche *et al.*, 2006) was calculated to evaluate SDMs skills. Once the six PFG-specific predictors were selected, the species presences-background data were randomly split into 10 different training and testing sets (80–20%, respectively). Therefore, 30 models (3 SDM types × 10-fold cross-validation) were trained per species. Model skill was evaluated through the TSS. All 845 species displayed an average TSS > 0.30, so they were all retained.

#### Projections of species richness

For each species, the 30 models were projected onto the 12 monthly climatologies of the predictors included in the SDMs, thus generating global projections of monthly habitat suitability indices (HSIs). The latter highlight the regions where environmental conditions are most favourable for a species to be present. Since the main goal of our study is to study species richness patterns, each model’s HSI projection was converted to a presence–absence distribution map based on the HSI threshold that maximized the TSS score of the model. For each PFG, the model-specific monthly maps were stacked to estimate their monthly richness and species composition. Annual mean richness was then derived by averaging monthly estimates for each model. Following an ensemble projection approach, the final ensemble projections of annual mean richness were obtained by averaging the model-specific annual richness estimates. True richness levels cannot be estimated here since many taxa that contribute to the richness of the present PFGs in nature cannot be modelled due to the limited occurrence density. Therefore, the final species richness estimates were divided by the number of species modelled in each PFG to present species diversity estimates that represent the emergent global gradients in PFGs diversity.

Because the species richness estimates were normalized to the number of species modelled, they range between 0 and 1 with values equal to 1 indicating the grid cells where environmental conditions allow all species to be modelled as present (i.e. species accumulation). Based on these estimates, we explored the inter-PFGs differences in latitudinal diversity gradients in three ways. First, we clustered the groups based on the similarity of their latitudinal diversity gradients, which was estimated through a Euclidean distance matrix. Then, we plotted this similarity matrix through a heatmap whose tiles are organized as a function of the dendrogram issued from the similarity matrix (Document S1). Second, we quantified the strength of the covariance between mean latitudinal richness and absolute latitude through rank correlation coefficients (*ρ*). This way, we identified which PFGs show a diversity gradient that can be predicted by latitude, whatever the strength of this gradient. Third, we fitted linear regressions between mean latitudinal species richness and absolute latitude. The linear regressions were not computed across the full latitudinal range, as most PFGs displayed non monotonic and non-linear latitudinal gradients of richness (Document S1). Here, we aimed to compare PFGs on the basis of their relative rate of species accumulation, rather than trying to prove the existence of linearity among the latitudinal gradients. To do so, we computed the first derivatives of the mean latitudinal annual richness over absolute latitude to detect the latitudinal ranges over which the groups’ richness shows a monotonic decrease in species richness (Document S1). Absolute latitudes > 65° were excluded since changes in diversity beyond this threshold only occur within a very small (often < 0.1) ranges of species richness. The linear regressions were fitted from the first absolute latitude that is followed by 10 consecutive negative derivative values (i.e. a monotonic decrease in richness) to the first latitude showing a positive derivative (i.e. richness increase). Two PFGs (Chaetognaths and Foraminifera; Document S1) showed a local maximum (i.e. 10 consecutive positive derivative values) between two phases of richness decrease. We chose to integrate these local maxima into the linear regressions instead of fitting two smaller and separate linear models for the same PFG. The slopes of the linear regressions were then used to compare the steepness of the latitudinal diversity gradient (i.e. the rate of species accumulation over latitude) between PFGs through covariance analysis (ANCOVA).

### Analyses

#### Similarity between richness patterns and their covariance with ecosystem properties

To evaluate the similarity between the patterns of annual richness across PFGs and help delineate their main modes of spatial variability, a principal component analysis (PCA; Legendre and Legendre, 2012) was performed on the ensemble estimates of mean annual richness. PCA is a dimensionality reduction analysis that summarizes the correlation structure between input variables through a symmetric covariance matrix whose ensuing eigenvectors are used to construct principal components (PCs).

We aimed to determine the main environmental covariates of species richness, so the annual climatologies of the following environmental predictors were added as supplementary variables in the PCA: SST, O_2_ (at 175-m depth), PAR, log(Chl-a), Wind, MLD, log(NO3), log(Si(OH)_4_), N^*^, Si^*^, log(EKE) and pCO_2_. Although they were not considered as candidate predictors for the SDMs, mean annual sea surface salinity (SSS) and the annual range of SST (dSST; maximum monthly value − minimum monthly value) were added in the PCA as supplementary variables to better represent the range of environmental conditions experienced by PFGs in the ocean. This way, we can examine the covariance between the PFGs-level richness and environmental predictors. We highlight that this is not a truly independent test as the monthly climatologies of some of these variables were used as predictors in the SDMs (see section Selection of Environmental Predictors).

Oxygen minimum zones (OMZs) and eastern boundary upwellings (EBUS) are major biogeochemical features of the ocean whose impacts on large-scale plankton diversity patterns remain relatively poorly studied (Chavez and Messié, 2009; Wishner *et al.*, 2018, 2020) compared to the impact of the global temperature gradient (Tittensor *et al.*, 2010; Benedetti *et al.*, 2021). Therefore, we also assessed the emergent impact of these two types of features on the species richness of the PFGs. To do so, we binarily classified (1/0) each grid cell of the ocean as an “OMZs cell” or an “EBUS cell” (see Document S2 for the full details) and performed two-sided Wilcoxon tests to examine whether OMZs and EBUS cells display higher or lower levels of PFGs SR compared to other cells from comparable latitudinal ranges.

Furthermore, we aim to examine the covariance between PFG species richness and five proxy variables of ecosystem functioning to investigate emergent biodiversity–ecosystem functioning relationships. The following five variables represent plankton-related processes or factors that provide key socio-economical services: (i) normalized global species richness of oceanic taxa (i.e. bony fishes, sharks, cetaceans and squids), which is indicative of overall marine megafauna biodiversity (Tittensor *et al.*, 2010); (ii) mean annual surface NPP (mg carbon m^−2^ day^−1^) (DeVries and Weber, 2017); (iii) the corresponding flux of particulate organic carbon (FPOC, mg carbon m^−2^ day^−1^) that is exported below the euphotic zone, which indicates the strength of the biological carbon pump (DeVries and Weber, 2017); (iv) the FPOC/NPP ratio (hereby called the e-ratio), which indicates the efficiency of the biological carbon pump; and (v) the inverse of the mean annual slope of the power-law particles size distribution measured from satellites (Kostadinov *et al.*, 2009), which serves as a quantitative particle size index (PSI; the higher the value, the higher the contribution of larger particles and phytoplankton cells to the total size spectrum). These five proxies of marine ecosystem services were also included in the same PCA as supplementary variables. This way, this single multivariate analysis enabled us to (i) identify the PFGs that display similar mean annual richness patterns; (ii) identify the main environmental gradients covarying with these species richness patterns; and (iii) explore the emerging patterns between the richness of various PFGs and proxy variables of ecosystem functioning.

#### Uncertainties in projections of mean annual PFGs species richness

The present global richness estimates are sensitive to sources of uncertainty, i.e. factors of the modelling framework, such as SDM type choice or species prevalence, that lead to inter-model variability in species richness projections (Thuiller *et al.*, 2019). Evaluating the uncertainty levels can help identify the PFGs for which current SDMs approaches provide less robust richness estimates. The standard deviation of the 30 model-specific annual richness projections was computed for each PFG to quantify their relative level of uncertainty in annual richness estimates. The linear relationship between this estimate of relative uncertainty and the number of species modelled and/or sampling effort per PFG (i.e. the number of occurrences available for training the SDMs) was examined to test if uncertainties in species richness estimates decrease with higher sampling effort.

## RESULTS

### Relative importance of environmental predictors for constraining species distributions

**Fig. 1:**
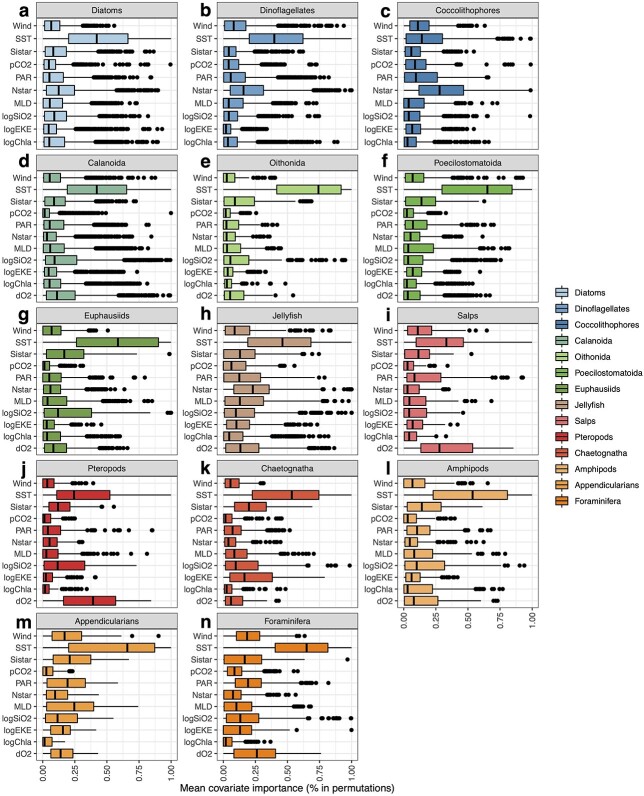
Distribution of the ranks of relative importance of each environmental predictor investigated for modelling the species distributions of (**a**) Diatoms (*n* = 163), (**b**) Dinoflagellates (*n* = 157), (**c**) Coccolithophores (*n* = 27), (**d**) Calanoida (*n* = 221), (**e**) Oithonida (*n* = 12), (**f**) Poecilostomatoida (*n* = 41), (**g**) Euphausiids (*n* = 51), (**h**) Jellyfish (*n* = 69), (**i**) Salps (*n* = 12), (**j**) Pteropods (*n* = 19), (**k**) Chaetognatha (*n* = 27), (**l**) Amphipods (*n* = 20), (**m**) Appendicularians (*n* = 6) and (**n**) Foraminifera (*n* = 26). For each predictor and species, ranks were determined through 30 random permutation tests for each SDM (30 models, so *n* = 90 scores in total). Ranks were then aggregated at the PFG level. The central vertical lines indicate the median values, the boxes illustrate the interquartile ranges and the error bars indicate 5th and 95th percentiles. The dots beyond the upper hinges of the boxplot represent outliers.

Overall, SST emerged as the top-ranking predictor constraining the species distributions (median rank ± IQR = 0.42 ± 0.51), phytoplankton and mixotrophic groups (0.35 ± 0.45) and zooplankton groups (0.47 ± 0.53). The rankings of the other predictors depended on the aggregation level. When taking all 851 plankton species together, SST was followed by O_2_ (zooplankton only), N^*^, Si^*^/log(Si(OH)_4_) (non-significant variations based on Dunn’s pairwise multiple comparison tests), Wind/PAR, MLD, log(EKE)/log(Chl-a) and pCO_2_, which was found the least important variable. For phytoplankton and mixotrophic species, SST was followed by N^*^, Wind, PAR/Si^*^/log(Si(OH)_4_) and the pCO_2_/MLD,/log(Chl-a)/log(EKE) group. For zooplankton species, SST was followed by: O_2_/Si^*^/log(Si(OH)_4_), PAR/Wind/MLD, log(EKE)/N^*^, log(Chl-a) and then pCO_2_ again as the least-performing predictor. SST remained the top predictor at the PFG level, except for coccolithophores and pteropods whose top-ranking predictors were N^*^ and O_2_, respectively (Fig. 1c and j). The top six ranking variables of each PFG that was retained to train the SDMs are given in Table S3.

### Global latitudinal gradients in mean annual species richness

**Fig. 2:**
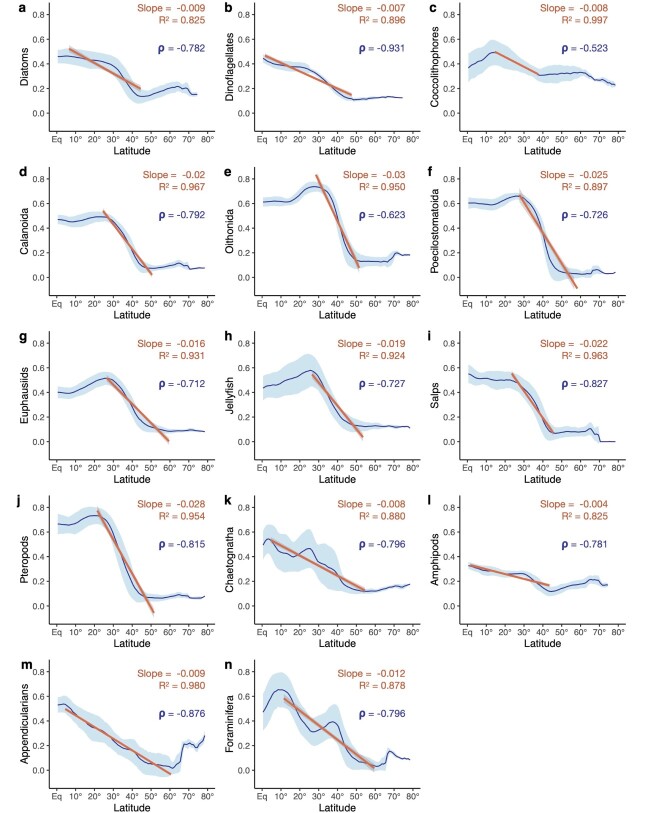
Zonal distribution of mean annual richness (expressed in % of species modelled per group) as a function of absolute latitude for (**a**) Diatoms (*n* = 163), (**b**) Dinoflagellates (*n* = 157), (**c**) Coccolithophores (*n* = 27), (**d**) Calanoida (*n* = 221), (**e**) Oithonida (*n* = 12), (**f**) Poecilostomatoida (*n* = 41), (**g**) Euphausiids (*n* = 51), (**h**) Jellyfish (*n* = 69), (**i**) Salps (*n* = 12), (**j**) Pteropods (*n* = 19), (**k**) Chaetognatha (*n* = 27), (**l**) Amphipods (*n* = 20), (**m**) Appendicularians (*n* = 6) and (**n**) Foraminifera (*n* = 26). The curves illustrate the latitudinal averages of species richness per absolute degrees of latitude. The lighter ribbons illustrate the standard deviation associated with the latitudinal averages (i.e. longitudinal variability). The solid lines illustrate the linear regressions computed over the latitudinal range of interest. The slope of the linear regressions indicates the strength of the decrease in species richness, and the adjusted *R*^2^ indicates the quality of the linear regressions. The Spearman’s correlation coefficients (*ρ*) indicate the strength of the covariance between absolute latitude and average species richness. All model coefficients and correlation coefficients show *P*-values < 0.001.

**Fig. 3:**
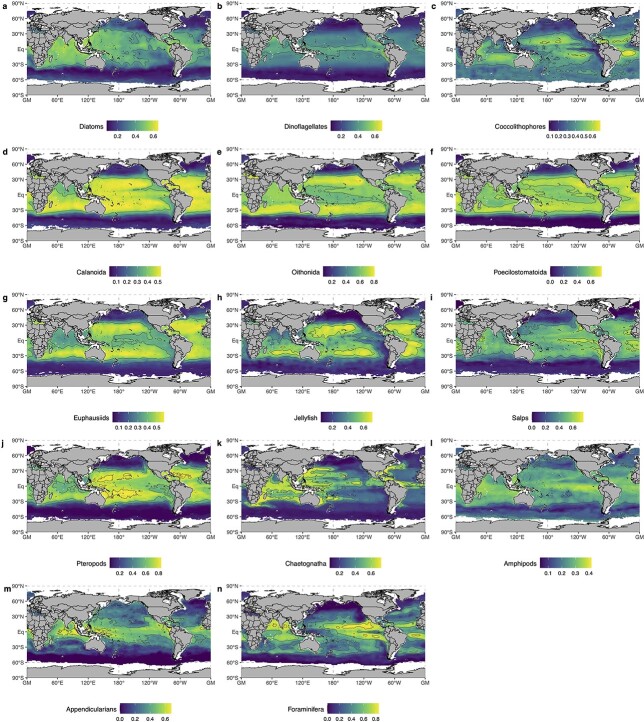
Spatial distribution of the mean annual species richness (expressed in % of species modelled per group) for (**a**) Diatoms (*n* = 163), (**b**) Dinoflagellates (*n* = 157), (**c**) Coccolithophores (*n* = 27), (**d**) Calanoida (*n* = 221), (**e**) Oithonida (*n* = 12), (**f**) Poecilostomatoida (*n* = 41), (**g**) Euphausiids (*n* = 51), (**h**) Jellyfish (*n* = 69), (**i**) Salps (*n* = 12), (**j**) Pteropods (*n* = 19), (**k**) Chaetognatha (*n* = 27), (**l**) Amphipods (*n* = 20), (**m**) Appendicularians (*n* = 6) and (**n**) Foraminifera (*n* = 26). The data used here are the same as in Fig. 2. Blank areas correspond to those grid cells where richness projections were not possible for more than 6 months due to missing values in the monthly environmental climatologies the models were projected on.

All PFGs display decreasing species richness from the low to the high latitudes, and all showed peaks in mean annual richness within the tropical band (0–30°) or near it (Figs 2 and 3; Document S1). Yet, PFGs show marked contrasts in their latitudinal diversity gradients as their richness showed varying strength in covariance with absolute latitude and varying rates of species accumulation (Fig. 2). Most groups showed strong negative correlations with absolute latitude, with *ρ* values ranging between −0.70 and −0.80 (all *P* < 0.001). Coccolithophores richness shows the weakest covariance with latitude (*ρ* = −0.52), whilst Dinoflagellates richness shows the strongest (*ρ* = −0.93). ANCOVA reveals that the coefficients of the linear regressions fitted vary significantly between PFGs (*F* = 1256.11; *P* < 0.001), indicating that most PFGs show different rates of decrease in species accumulation along the monotonic parts of the richness gradients. Post hoc Tukey’s multi-comparison tests confirm that most pairs of PFGs show significant differences in the slope of the linear models (adjusted *P* < 0.001; Document S1). Overall, Oithonids, Pteropods, Poecilostomatoids and Salps show the steepest latitudinal diversity gradients (slopes < −0.02). Meanwhile, Amphipods, Appendicularians, Chaetognaths, Diatoms, Dinoflagellates and Coccolithophores show the weakest latitudinal diversity gradients (slopes > −0.004). The phytoplankton and mixotrophic groups tend to show weaker slopes and therefore weaker rates of decrease in richness than most of zooplankton groups. Among the zooplankton, Pteropods, Salps, Jellyfish and the three copepod groups show the strongest weaker rates of decrease in richness from the tropics to the high latitudes. In the tropical band, all PFGs show mean annual richness values above or close to 0.40 except Amphipods (mean ± SD = 0.28 ± 0.02). In the polar areas (>60°), all PFGs show mean diversity values > 0.10 except the Calanoids, Poecilostomatoids, Euphausiids and Salps.

The spatially explicit patterns of global PFGs richness are given in Fig. 3 to illustrate the longitudinal variability (i.e. light-blue ribbons on Fig. 2) on top of the main latitudinal patterns. Correlation coefficients were computed between species richness and longitude (spanning 0–360°) to test for potential longitudinal patterns as well. All PFGs display significant (all *P* < 0.01) but quite weak (all |*ρ*| < 0.2) longitudinal patterns of richness. Since these correlation coefficients are much weaker than those based on latitude (Fig. 2), we choose not to comment them further. The maps also highlight the relative position of the peaks and dips in mean annual richness of each functional group (but see section Emergent Relationships between Species Richness and Environmental Covariates for further description).

### Emergent relationships between species richness and environmental covariates

**Fig. 4:**
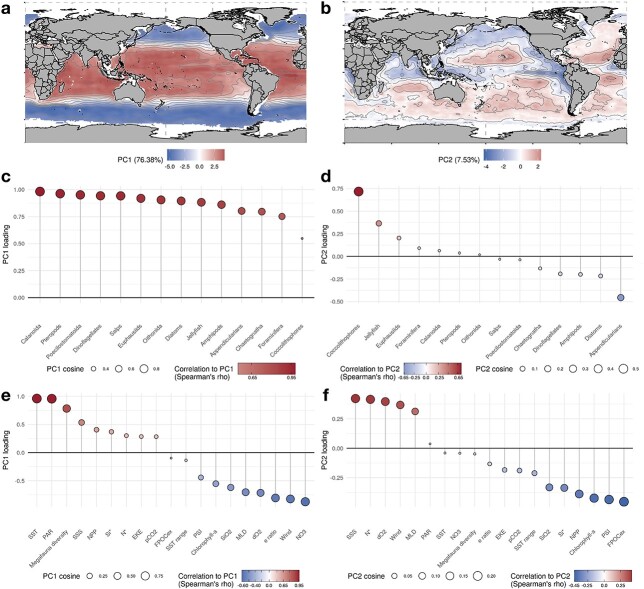
Projections of the first two components of a principal component analysis (PCA) based on the ensemble projections of mean annual species richness of the PFGs for the global surface open ocean (*n* = 29 680 ocean grid cells). Maps (**a**) and (**b**) show the spatial variability of PC1 and PC2, respectively. The lollipop plots in (**c**) and (**d**) indicate the loadings of the groups’ annual species richness that were used to construct PC1 and PC2. The lollipop plots in (**e**) and (**f**) indicate the loadings of the mean annual climatologies of environmental predictors that were added as supplementary variables in the PCA to analyse their covariance with PC1 and PC2. In the lollipop plots, the circles are coloured as a function of the variables’ correlation to the PCs. The size of the circles indicates the quality of the variables’ projection in the PCA space based on their squared cosine.

A PCA identified the equatorward increase in richness to be the first-order pattern as PC1 summarizes nearly 77% of the spatial variance and all PFGs scored positively along PC1 (Fig. 4a and c). Whilst PC2 explains a lower amount of variance (7.5%, Fig. 4b) it allows us to distinguish PFGs based on the location of their relative peaks and troughs in mean annual richness, permitting us to investigate productivity-related patterns that are decoupled from the global temperature gradient. PC2 is mainly scored by the species richness of Coccolithophores, Jellyfish and Euphausiids on the positive side and Appendicularians, Amphipods, Diatoms and Dinoflagellates on the negative side (Fig. 4d). Consequently, the regions in blue on Fig. 4b correspond to those regions where the latter four PFGs have peaks in species richness, whereas the regions in red indicate those where the richness of Coccolithophores, Jellyfish and Euphausiids peak.

The PCA also allows us to uncover the combination of environmental variables that covary most strongly with the patterns of species richness, including those predictors that were not used in the SDMs (Fig. S4). The environmental covariates that display a strong latitudinal gradient on a mean annual scale display the strongest covariation with PC1: SST and PAR with positive correlations and log(NO3), log(Si(OH)_4_), Wind, O_2_, MLD and log(Chl-a) with negative correlations (Fig. 4e). Then, the environmental variables that covary most strongly with PC2 are SSS, N^*^, O_2_, Wind and MLD on the positive side *versus* log(Chl-a), log(Si(OH_4_)) and Si^*^ on the negative side (Fig. 4f). Therefore, the PCA indicates that the global gradient of temperature- and productivity-related variables constitutes the first-order covariate of the PFGs’ species diversity. Meanwhile, the second-order covariate encapsulates gradients in nutrient ratios, oxygen concentration at depth, phytoplankton biomass and water mixing that separate oligotrophic gyres from upwelling systems, western boundary currents and minimum oxygen zones (Fig. 4b and f). This second-order gradient covaries with the mean annual richness of Coccolithophores, Jellyfish, Euphausiids, Appendicularians, Diatoms and Amphipods the most (Fig. 4d).

The PCA also allows us to investigate emergent biodiversity–ecosystem functioning relationships as proxies of marine ecosystem services were also included as covariates. These proxies covary with the two PCs with varying strengths. Annually integrated megafauna diversity and mean annual net primary production (NPP) were positively correlated to PC1, whereas the e-ratio (i.e. efficiency of the biological carbon pump) and the plankton size index (PSI) were negatively correlated with PC1. The mean annual FPOC, the PSI, chlorophyll-a concentration and NPP were the proxies scoring PC2. Therefore, regions in blue on Fig. 4b indicate regions where the plankton communities are associated with productive conditions, larger phytoplankton cells and high POC fluxes at depth.

### Uncertainties

**Fig. 5:**
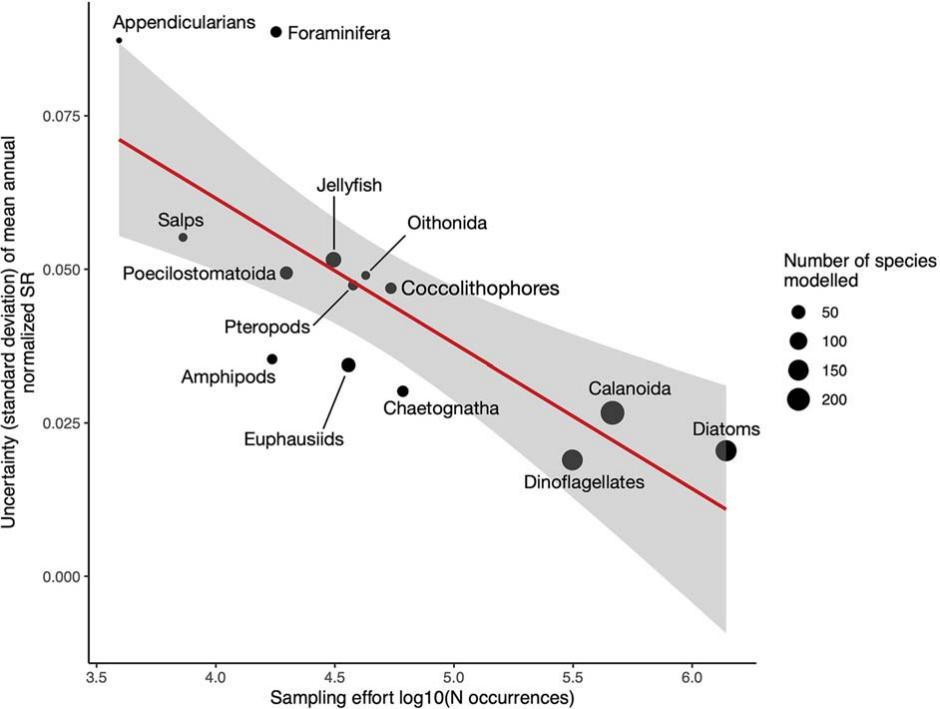
Negative linear relationship between sampling effort expressed as the logged (log base 10) number of species-level occurrences per PFG and the median uncertainty of the ensemble estimates of mean annual species richness (expressed in % of species modelled per group). Global scale species richness uncertainty was estimated as the standard deviation of the mean annual richness across ensemble model members (*n* = 30). The values shown here correspond to the median of the PFG-level uncertainty for the global ocean. The size of the points shows the number of species modelled per PFG. The semi-transparent grey ribbon illustrates the standard error interval of the linear regression (*n* = 14, adjusted *R*^2^ = 0.55, intercept = 0.15, slope = −0.01; *P*-value = 0.001).

Global mean uncertainty levels (Fig. S3) showed significant variations across PFGs (Kruskal–Wallis rank sum test, chi^2^ = 156 454, *P* < 0.001). Most pairwise tests of uncertainty variations between PFGs (post hoc Dunn’s tests with a Bonferroni method for *P*-value corrections) showed significant variations (*P* < 0.001), except the following pairs (all *P* > 0.01): Coccolithophores × Poecilostomatoids, Jellyfish × Oithonids, Salps × Jellyfish and Salps × Oithonids. Two PFGs have clearly higher levels of mean uncertainties in global richness estimates (Fig. 5): Foraminifera (0.09 ± 0.06) and Appendicularians (0.08 ± 0.04). On the opposite end, Dinoflagellates, Diatoms and Calanoids show much lower mean uncertainty levels (all median uncertainties < 0.03). A strong negative linear relationship was found between uncertainty levels and sampling effort (Fig. 5) indicating that uncertainty levels in mean annual richness decrease as more observations are available for fitting the SDMs. The mean uncertainty levels of Foraminifera and Appendicularians are above the standard error interval of the linear regression, suggesting the uncertainties of their richness estimates were higher than what can be predicted by sampling effort alone. On the opposite, Chaetognaths, Euphausiids and hyperiid Amphipods showed uncertainty levels lower than what can be predicted from sampling effort. Average uncertainty also decreased with the number of species modelled per group, but the linear fit was poorer than when considering sampling effort (adjusted *R*^2^ = 0.33, intercept = 5.75 × 10^−2^, slope = −1.93 × 10^−4^; *P* = 0.02).

## DISCUSSION

### Patterns and drivers of latitudinal diversity gradients across PFGs

With their increasing species richness from the high latitudes to the low latitudes (Figs 2 and 3), all 14 modelled PFGs show latitudinal diversity gradients that are typical of marine ectotherms (Tittensor *et al.*, 2010; Beaugrand *et al.*, 2015). Perhaps even more notable is that none of the investigated groups shows a reverse gradient (Fig. 2). This suggests that the underlying processes that control species diversity are common to widely different functional groups of plankton. Although all PFGs show decreasing diversity from the low to the high latitudes, they also show marked contrasts in the rate at which species accumulate with latitude (Fig. 2). Pteropods, Poecilostomatoids, Oithonids and Salps show the steepest latitudinal diversity gradients, whilst Amphipods, Appendicularians and the three phytoplankton groups show the weakest ones. The second component of the PCA (Fig. 4b, d and f) captures the richness patterns that modulate those differences in latitudinal gradients (Fig. 4a, c and e), permitting us to better analyse the covariance of PFG species richness with productivity-related variables (i.e. N^*^, Si^*^, chlorophyll-a, NPP; Fig. 4f).

The species diversity of Diatoms and Dinoflagellates show a stronger covariance with latitude than Coccolithophores diversity (Fig. 2). Plus, the diversity of the latter decreases over a narrower latitudinal range (Fig. 2; Document S1). Coccolithophores diversity is lower in eastern boundary upwelling systems and peaks in oligotrophic gyres (Fig. 3c). Meanwhile, the latter emerge as more favourable regions for Diatoms and Dinoflagellates diversity, whose species richness dips in the high latitudes contrary to Coccolithophores (Fig. 4b and d). This distinction stems from the way the SDMs captured the relationships between the occurrence data and the environmental predictors: Coccolithophores are the only PFG whose top ranking predictor was N^*^ instead of SST (Fig. 1), so the emergent Coccolithophore diversity patterns were less related to the latitudinal SST gradient compared to other PFGs (Figs 3 and 4). As a result, the strongest environmental covariates of Diatoms and Dinoflagellates diversity differ from those of Coccolithophores diversity (Fig. 4f). Coccolithophores richness is positively associated with N*, i.e. the excess of nitrate over phosphate, and negatively associated with Si*, i.e. the excess of silicic acid over nitrate. The silicifying Diatoms show the opposite pattern as they are known to benefit from high silicic acid concentrations (Sarthou *et al.*, 2005; Endo *et al.*, 2018). We interpret this difference between Coccolithophores and Diatoms/Dinoflagellates as an emergent pattern stemming from their distinct ecological strategies and physiological traits. Most Coccolithophores (except species like *Emiliania huxleyi*; Paasche, 2001) are considered K-strategists characterized by higher nutrient affinity and lower growth rates that enable them to prevail in stable and oligotrophic conditions. Conversely, Diatoms and Dinoflagellates are usually considered as r-strategists that thrive in nutrient-replete and turbulent conditions thanks to their higher growth rates (Margalef, 1978). Such contrasting diversity patterns may emerge on a macroecological scale if Diatoms species recurrently outcompete Coccolithophores species under conditions of silicic acid replenishment, whilst being outcompeted under the conditions that lead to non-Redfield ratio uptakes of nitrogen (and higher N* values; Martiny *et al.*, 2013). Therefore, the imprint of the distinct traits and ecological strategies on the biogeography (meaning the relationship between the species occurrences and the environmental predictors) of Coccolithophores and Diatoms/Dinoflagellates may be so strong that it was captured by our modelling approach. In situ observations (Smith *et al.*, 2017; Endo *et al.*, 2018) and ecosystem modelling studies (Nissen *et al.*, 2018) support such resulting strong zonation of these two phytoplankton functional groups.

Among zooplankton groups, inter-groups differences along PC2 are less marked (Fig. 4f) as most of these PFGs show similar latitudinal gradients in richness that mainly covary with temperature-related factors (Fig. 4c and e). The strongest differences in mean annual richness are found between Jellyfish and Euphausiids versus Appendicularians and Amphipods. The former group has its highest species diversity in the subtropical gyres, whilst their richness dips in eastern upwelling systems and areas characterized by the presence of OMZs at depth. In contrast, the species richness of the Appendicularians and Amphipods peaks in eastern upwelling systems and OMZs (Fig. 3; Document S2). Amphipoda species richness also sits on the negative side of PC2 because it is not minimal in the Southern Ocean (Figs 2–4). Most of the Amphipods modelled here belong to genera that mainly occur in high-latitude ecosystems such as the Southern Ocean or the Arctic (e.g. *Themisto* spp.; Havermans *et al.*, 2019). Therefore, we are confident that the relatively high mean annual richness of Amphipods at high latitudes reflects the imprint of the environment on their biogeography, likely due to the environmental filtering of their peculiar traits (but see Havermans *et al.*, 2019). Most of the Amphipods modelled are known commensals and parasitoids of gelatinous plankton such as Jellyfish (Burridge *et al.*, 2017), so their non-overlapping richness peaks along PC2 may seem surprising. It should be noted that most of the occurrences used here come from traditional plankton sampling techniques that are known for breaking the soft bodies of gelatinous zooplankton such as medusae and ctenophores. Therefore, our approach is unlikely to capture the imprint of fine-scale parasitic relationships between gelatinous zooplankton and Amphipods on their species distribution and thus their large-scale species richness patterns.

The Appendicularians stand out because their species richness peaks in areas characterized by OMZs (Fig. 3m; Document S2), a pattern that is hard to interpret because Appendicularians are not known to be particularly affiliated to such conditions. The recent global study by Soviadan *et al.* (2022) found pelagic tunicates (i.e. Salps and Appendicularians) to be more tolerant to the presence of OMZs than other zooplankton, in line with previous observations (Thuesen *et al.*, 2005; Trueblood, 2019). We find oxygen to be an important predictor for Salps distribution (Fig. 1), and we find Salps diversity to be weakly affected by the presence of OMZs contrary to all other zooplankton groups (Document 2), which could be a reflection of their relatively high tolerance to low oxygen conditions. Meanwhile, other PFGs that are known to be hypoxia-tolerant, like Jellyfish, Foraminifera, Oithonids and Poecilostomatoids (Thuesen *et al.*, 2005; Hauss *et al.*, 2016; Soviadan *et al.*, 2022), show lower richness in OMZs (Document S2). We put forward two hypotheses to explain such discrepancy: (i) method limitation, i.e. our approach may be limited in how well it can capture the imprint of low-oxygen tolerances on the group-level biogeographies between Salps and other groups (because of group-level differences in data coverage, for instance); (ii) species-level differentiation, i.e. the emergent patterns are driven by species-level differences in oxygen tolerances within the groups themselves (e.g. not all Jellyfish species are equally tolerant to low oxygen concentrations), which may lead to decreases in species-level habitat suitability indices and thus a decrease in richness in areas where OMZs occur.

The drivers of plankton community assembly and diversity are debated as various studies identified different drivers, depending on whether they relied on traditional observations, metagenomics or theoretical models (Dutkiewicz *et al.*, 2020; Benedetti *et al.*, 2021;Richter *et al.*, 2021; Sommeria-Klein *et al.*, 2021). Although our statistical approach does not allow to pinpoint the ecological mechanisms that determine the inferred latitudinal diversity gradients, the PFG-level ranks of relative importance (Fig. 1) and the covariance analysis (Fig. 4 and S4) support the view that temperature and the environmental factors covarying with it are the first-order controls on the distribution of species richness of the PFGs on a global scale. Therefore, on the one hand, our findings lend support to the kinetic energy hypothesis and the metabolic theory of ecology, according to which higher temperatures promote higher species diversity through increased speciation rates and the selection of warm-water-tolerant species (Tittensor *et al.*, 2010; Brown, 2014; Benedetti *et al.*, 2021). On the other hand, trait-based ecosystem models found that various dimensions of global phytoplankton diversity are rather controlled by the rates of resource supplies, the grazing-mediated selection of functional types and the turbulence associated with mesoscale and sub-mesoscale activity (Barton *et al.*, 2010; Dutkiewicz *et al.*, 2020). Other observational studies based on high-throughput DNA sequencing found local abiotic and biotic (e.g. biological interactions) factors and current-mediated dispersal to explain latitudinal patterns in plankton community structure (Richter *et al.*, 2021; Sommeria-Klein *et al.*, 2021). Here, environmental predictors related to climatic variability and turbulence at the scale of our study display either no correlations (e.g. annual SST range and EKE) or negative ones (e.g. MLD and wind speed) with the PFGs’ mean annual richness (Figs 4 and S4). In addition, MLD, wind speed and EKE do not surpass SST in terms of predictors’ rankings (Fig. 1). The overall lack of positive relationships between mixing-related factors and species richness does not fall in line with the model-based studies that suggest that mixing and mesoscale activity promote the co-existence and evenness of PFGs (Barton *et al.*, 2010; Dutkiewicz *et al.*, 2020; Mangolte *et al.*, 2022). These studies were conducted at an eddy-permitting (or eddy-resolving; Mangolte *et al.*, 2022) resolution and incorporate competition processes between size-based PFGs, but are rarely evaluated against in situ data. Meanwhile, our approach is based on observations aggregated throughout decades to constrain empirical SDMs on spatial and temporal scales (1 × 1, monthly) that do not allow to resolve such competition effects, nor the impact of mesoscale features.

As already shown in terrestrial ecology, the imprint of various biological, ecological and climatic processes on species diversity is strongly scale dependent (Thuiller *et al.*, 2015; Gonzalez *et al.*, 2020). Therefore, we suggest that the discrepancies highlighted above may be related to the scale-dependency of the various processes that structure marine plankton biodiversity. Different abiotic and biotic factors may emerge as dominant drivers of marine plankton species richness depending on the spatial and temporal scales covered by the data at hand.

### Emergent biodiversity–ecosystem functioning relationships

We find that the species richness of PFGs covaries positively with the species richness of oceanic fishes, sharks and mammals (Fig. 4e and f), which implies that the diversity of PFGs and higher trophic levels are shaped by similar temperature-related drivers (Tittensor *et al.*, 2010). This further supports the realism of our richness estimates as our SDMs ensemble seems to capture the main processes that constrain marine biodiversity on a global scale. Yet, we cannot here establish how the diversity of the smaller plankton influences the diversity of higher trophic levels. Second, we find that the efficiency of the biological carbon pump (i.e. the e-ratio) decreases with the species richness of all PFGs (Fig. 4g and h). This finding is in line with the view that species-rich and functionally diverse communities are better able to retain the biomass produced within surface layers, whereas species-poor communities tend to leak higher rates of biomass out of the surface ocean. In addition, the PSI shows very similar correlation patterns to the e-ratio, which implies that the emergent negative relationship between plankton species diversity and the efficiency of the biological carbon pump could also be mediated by changes in size structure. Indeed, the species-rich communities from the tropics tend to be composed of smaller species compared to the species-poor communities from the high latitudes (Uitz *et al.*, 2010; Acevedo-Trejos *et al.*, 2015, 2018; Brandão *et al.*, 2021). High-latitude communities tend to have higher proportions of large phytoplankton cells and large-bodied zooplankton whose functional traits are known to favour faster export of POC to depth (Stamieszkin *et al.*, 2015; Tréguer *et al.*, 2018; Brun *et al.*, 2019; Henson *et al.*, 2019). However, here, the absolute quantity of POC exported below the euphotic zone is covarying with PC2 and not PC1, meaning that it does not show strong correlation with the global species diversity gradients of most PFGs. It could be that the diversity of zooplankton groups does not strongly control the total amount of POC export but rather the fraction of NPP that is turned into export fluxes (Henson *et al.*, 2019). More research is needed to help disentangle the effects of species richness from those of size distribution (or other size-related traits; Litchman *et al.*, 2013) on export production and its efficiency.

The relationship between primary production and zooplankton diversity is unclear. Few zooplankton groups show strong loadings on PC2, which captures the global productivity gradient (Fig. 4) and because log(Chl-a) ranks among the least important predictors of zooplankton species ranges (Fig. 1; Table S3), meaning it was not included as a predictor in most of the SDMs. Although phytoplankton biomass is a determining factor for zooplankton biomass (Strömberg *et al.*, 2009; Drago *et al.*, 2022; Knecht *et al.*, 2023), it could be less relevant for determining the spatial ranges of zooplankton species, and thus emergent species richness, if those are mainly controlled by temperature (Fig. 1; Benedetti *et al.*, 2021). We examined the potential link with productivity by testing whether the PFGs show higher or lower mean annual richness within EBUS, which represent relatively warm and productive areas (Chavez and Messié, 2009), compared to other tropical and less productive areas (Document S2). We find that most PFGs (except Salps, Amphipods and Poecilostomatoids) show significantly lower species richness in EBUS. This is interesting as it implies that conditions of higher productivity are not favourable to all species within PFGs, assuming that most zooplankton groups rely on primary production either directly (i.e. through grazing or filter feeding of particles) or indirectly (i.e. through the predation smaller grazing zooplankton). This finding supports the view that such conditions lead to environmental and biotic filtering (i.e. competitive exclusion) that select a subset of taxa at the expense of others, likely because of fitter traits combinations (Falkowski and Oliver, 2007; Litchman *et al.*, 2010). Conversely, it contradicts the view that niche partitioning promotes the co-existence of various species within PFGs. As the phytoplankton and mixotrophic groups show lower species richness in EBUS (Document S2) and in productive temperate areas characterized by seasonal bloom regimes (Fig. 4; Righetti *et al.*, 2019), our results suggest that, at the scale of our study, higher productivity might be carried by subsets of the autotrophic and mixotrophic protists community.

Ultimately, the richness of phytoplankton groups decreases with higher nutrient availability, which can be interpreted through two mutually non-exclusive processes. On the one hand, species-rich communities draw down nutrient concentrations more efficiently, meaning that phytoplankton diversity optimizes nutrients use efficiency in the system (Falkowski and Oliver, 2007; Marañón, 2015). This would translate into lower relative rates of biomass exported from surface ecosystems, which is corroborated by the emergent negative correlations between phytoplankton species richness and the e-ratio (Fig. 4). On the other hand, systems such as tropical gyres that are characterized by weaker seasonal variations and lower nutrient availability might sustain species-rich communities through niche partitioning that enables the co-existence of many taxa characterized by different functional traits (Litchman *et al.*, 2010; Edwards *et al.*, 2016). Yet again, we underline that the scale of our study may not align with the finer spatio-temporal scales at which such processes occur, thus masking their influence. Under either hypotheses, our results point towards the existence of links between key ecosystem functions (i.e. nutrients use efficiency and carbon export) and the species diversity of PFGs. Our results warrant further field studies examining the relationships between ecosystem functions, species richness and community traits expression, as well as the verification and extension of existing ecological theory (Chust *et al.*, 2018).

### Uncertainty sources and caveats

In this study, PFGs had to be defined based on our current knowledge of the ecological and biogeochemical functions associated with each broad taxonomic group, as an exhaustive compilation of functional trait across multiple plankton species is still missing. One of the main caveats of this approach is that we are not accounting for species-level and organism-level variations in functional traits within PFGs. Within groups such as Diatoms, Jellyfish or Salps, species’ sizes span one or several orders of magnitude (Lucas *et al.*, 2011; Henschke *et al.*, 2016; Leblanc *et al.*, 2018). Since most functional traits scale with size (Litchman *et al.*, 2013), our approach underestimates the existence of the smaller functional groups nested within those chosen here (Leblanc *et al.*, 2018; Benedetti *et al.*, 2022). Recent functional trait syntheses enabled to identify at least 11 functional groups of planktonic copepods nested within the three groups used here (Benedetti *et al.*, 2022). Among the copepod groups studied here, the Oithonida constitute a homogeneous functional group but Calanoida and the Poecilostomatoida can be further divided into smaller groups depending on their feeding mode, size range or preferential food sources (see Benedetti *et al.*, 2022 for more details). Beyond cell and body size, some of the protist groups studied here are known to be mixotrophic rather than simply autotrophic or heterotrophic (Dinoflagellates and Foraminifera; Mitra *et al.*, 2023). Therefore, our simplifying classification does not disentangle such large groups into the various mixoplankton types that are nested within them (e.g. constitutive vs. non-constitutive mixoplankton; Mitra *et al.*, 2023). Based on the observations synthetized by Mitra *et al.* (2023) and Schneider *et al.* (2020), we indicate that most (45.5%) of the Dinoflagellates species modelled are constitutive mixoplankton that possess the innate biological machinery to perform photosynthesis (*Tripos* spp., *Prorocentrum* spp., *Gonyaulax* spp., *Gyrodinium* spp.; Table S1). Some (14.3%) are specialized non-constitutive mixoplankton (*Dinophysis* spp., *Podolampas* spp., *Phalacroma* spp., *Ornithocercus* spp.), whilst the remaining 40% remain described as “protozooplankton” that could be purely heterotrophs but also constitutive or non-constitutive mixoplankton (*Protoperidinium* spp., *Oxytoxum* spp., *Diplopelta* spp., etc.). Therefore, we believe that the present Dinoflagellates species richness estimates are mostly representative of the constitutive mixoplankton taxa. Unfortunately, more detailed studies similar to Benedetti *et al.* (2022) remain impossible to perform across more than 10 PFGs as long as we are lacking a “pan plankton” synthesis of functional traits for phyto- and zooplankton groups (Barton *et al.*, 2013; Litchman *et al.*, 2013; Martini *et al.*, 2021). This caveat is more relevant for those PFGs characterized by large species richness (*n* > 50; i.e. Dinoflagellates, Diatoms, Calanoids, Jellyfish and Euphausiids) as smaller PFGs are likely to display a much lower variability in traits. Our definition of PFGs aims to achieve an optimal trade-off between established inter-groups differences in size classes and ecological or biogeochemical functions (Le Quéré *et al.*, 2005; Hood *et al.*, 2006; Heneghan *et al.*, 2020). We are confident that the present PFGs capture distinct functional entities, at least from the inter-group point of view.

The other caveats of our approach are those that are inherent to the use of SDMs (Elith and Leathwick, 2009; Thuiller *et al.*, 2019). SDMs do not incorporate some of the processes that shape plankton distributions, such as dispersal, biotic interactions and population-level variations in niches (Elith and Leathwick, 2009). The present SDMs assume that plankton species distributions can be reproduced as a function of the combinations of environmental predictors defining a species’ niche. Such assumptions remain supported on macroecological scales where the imprint of biological interactions is considered to be relatively small (Araújo and Rozenfeld, 2014; Thuiller *et al.*, 2015) and where connectivity through sea currents is high enough to support weak dispersal limitations (Jönsson and Watson, 2016). There is now mounting evidence that the spatial ranges of marine ectotherms are mainly shaped by their thermal niches (Pinsky *et al.*, 2019; Fredston *et al.*, 2021) and that empirical distribution models robustly reproduce the distributions of marine ectotherms (Sunday *et al.*, 2012). As our ensemble of SDMs can reproduce the latitudinal diversity gradients observed for several groups, we are confident in the present estimates of mean annual richness (Benedetti *et al.*, 2021; Righetti *et al.*, 2023).

Nonetheless, we carefully investigated the level uncertainties associated to the latter (Figs 5 and S3). Previous work showed that SDMs choice drives the variability between model projections (Benedetti *et al.*, 2017; Thuiller *et al.*, 2019). We show that there are strong differences in uncertainty levels between PFGs, with Foraminifera and Appendicularia showing the most uncertain species richness estimates and Diatoms, Dinoflagellates and Calanoids showing the least uncertain (Fig. 5). Consequently, we believe that the present richness patterns should be taken with caution for Foraminifera and Appendicularia. PFG-level variations in species richness uncertainty could be robustly explained by differences in data coverage and species richness (Fig. 5). We acknowledge that these two factors are not independent since richer PFGs tend to have a larger pool of occurrences available for model training (Fig. S2). The diversity estimates of Foraminifera and Appendicularia (Figs 2 and 3) can thus be largely improved through our approach by enriching the spatial coverage of their occurrence pool, which is currently relatively low. A wealth of Foraminifera species occurrences derived from sediment records is available (Siccha and Kucera, 2017), but they could not be included in our study as we focus on samples taken in the upper water column to be consistent across all PFGs. Several studies used such sediment records to estimate pre-industrial and modern planktic Foraminifera diversity gradients (Lombard *et al.*, 2011; Jonkers *et al.*, 2019; Yasuhara *et al.*, 2020). They usually found Foraminifera diversity to peak polewards of the subtropics (~40° latitude). Therefore, the fact that we find Foraminifera richness to peak between 0° and 20° latitude could be driven by limited data availability.

We underline that we aimed to model species richness gradients and not absolute species richness values since many species could not be modelled due to limited occurrence numbers. Here, we managed to model ~20% of the 1704 species recorded in PhytoBase (Righetti *et al.*, 2020) and 15% of the 3206 species recorded in ZooBase (Benedetti *et al.*, 2021). As a consequence, some of the present richness patterns are likely underestimated in regions of the ocean where lower sampling effort may have led to incomplete sampling of the plankton community (Fig. S2). The species modelled here are those that are the most frequently detected by traditional sampling and identification techniques either because they dominate the community in terms of abundance and biomass or because they correspond to relatively large species that are more easily sampled and identified by taxonomists (Table S1). Ser-Giacomi *et al.* (2018) showed that the ranges of rare and non-dominant plankton species exhibit no biogeographical signature, meaning that they are hardly relatable to environmental gradients. Consequently, there is no guarantee that such rare taxa can be robustly modelled through our numerical approach. Nevertheless, the recent study by Righetti *et al.* (2023) showed that the SDM framework used here is able to robustly reproduce observed global species richness gradients from scattered data. Finally, the present diversity patterns are based on species richness (Figs 2 and 3) and not an abundance-weighted diversity index (e.g., Shannon–Wiener index, like in Barton *et al.*, 2010). Therefore, we are likely overestimating true diversity in areas where a subset of species dominates community abundance and biomass. The steepness of some of the present latitudinal gradients may be (i) underestimated if diversity is overestimated towards high latitudes or (ii) overestimated if diversity is overestimated in the tropics and near the equator.

## CONCLUSIONS

Our study provides new estimates of global ocean plankton species richness and composition obtained in a coherent and comparable manner based on state-of-the-art distribution modelling across 14 PFGs. We find that all PFGs display global diversity gradients that are likely driven by processes related to the latitudinal temperature gradient. This leads to richer communities in the low latitudes compared to the high latitudes, similar to what is observed for higher trophic levels (Tittensor *et al.*, 2010). Yet, individual PFGs show substantial differences in terms of the detailed pattern in annual mean species richness, with some groups having their maxima at the equator and others near 30° latitude. We interpret this to be a consequence of inter-group or intra-group competition and/or selection effects that imprint the biogeography of the individual PFGs. We suggest that the dominant processes underlying marine plankton diversity structure are scale-dependent, with temperature playing an important role on macroecological scales (e.g. planetary scale, basin scale, decadal scale), whilst other factors such as biotic interactions and dispersal may dominate on smaller scales (e.g. subregional to local scales, weekly scales).

Our study also supports the existence of links between ecosystem functions and the diversity of PFGs. We find that the efficiency of the biological carbon pump covaries negatively with plankton species richness, supporting the view that species-rich and functionally diverse communities are better able to recycle the biomass and nutrients within surface layers. Most PFGs show lower diversity in conditions of higher productivity, as those emerged as not favourable to all species within each PFG. Our study thus supports the view that biotic filtering (i.e. competitive exclusion) takes place in productive conditions as a subset of taxa are selected likely because of fitter traits combinations.

We hope that our study will be used as a basis to bridge the gap between observational and model-based studies and to identify the scaling laws that describe which drivers of plankton diversity govern community assembly. The development of modern imaging and DNA sequencing techniques will allow to get more complete listings of plankton species and their associated traits (de Vargas *et al.*, 2015; Sunagawa *et al.*, 2015), yet data availability is still too limited to investigate the scale dependence of the drivers of diversity and community assembly. Understanding how these new data types can be integrated in the present modelling framework and into new macroecological theories will be determinant to build a unifying and holistic vision of marine plankton biodiversity and its effects on ecosystem functions (Cardinale *et al.*, 2012; Gonzalez *et al.*, 2020).

## Supplementary Material

Figure_S1_fbad044

Figure_S2_fbad044

Figure_S3_fbad044

Figure_S4_fbad044

Table_S1_fbad044

Table_S2_fbad044

Table_S3_fbad044

## Data Availability

All the plankton species occurrences underlying the main results of this paper are publicly available online. See https://doi.pangaea.de/10.1594/PANGAEA.904397 for the phytoplankton data. See https://zenodo.org/record/5101349#.YSUOjMY69pQ for the zooplankton data. All R codes used to generate the results of the study are stored on the GitHub account of F.B (https://github.com/benfabio).
