# Supplementary figures and images for: Global gradients in species richness of marine plankton functional groups
Source: J Plankton Res. 2023 Oct 12;45(6):832–52. doi: 10.1093/plankt/fbad044 (PMC10710906; doi:10.1093/plankt/fbad044)

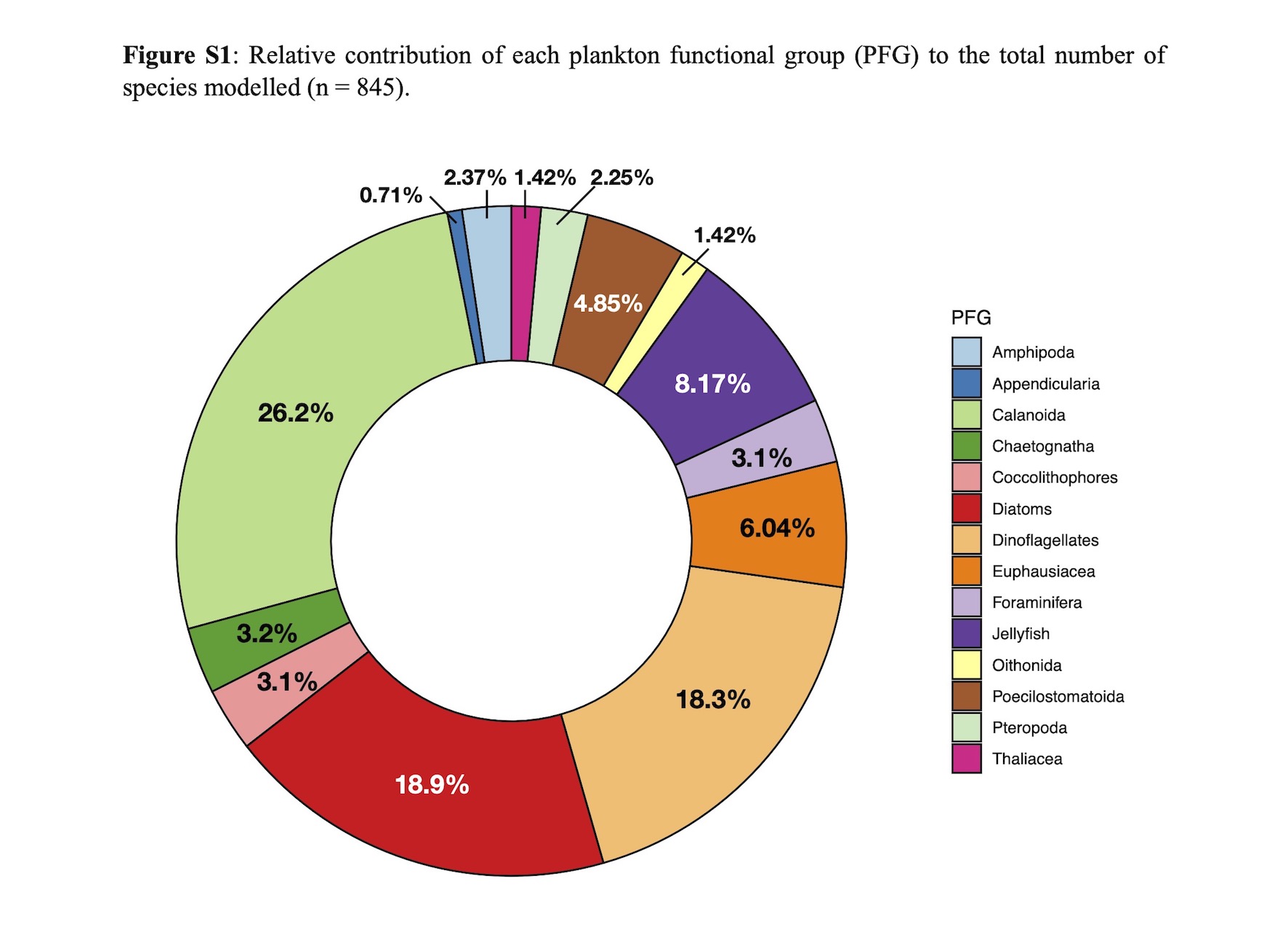

Supplement: Figure_S1_fbad044 [file figure_s1_fbad044.jpeg]

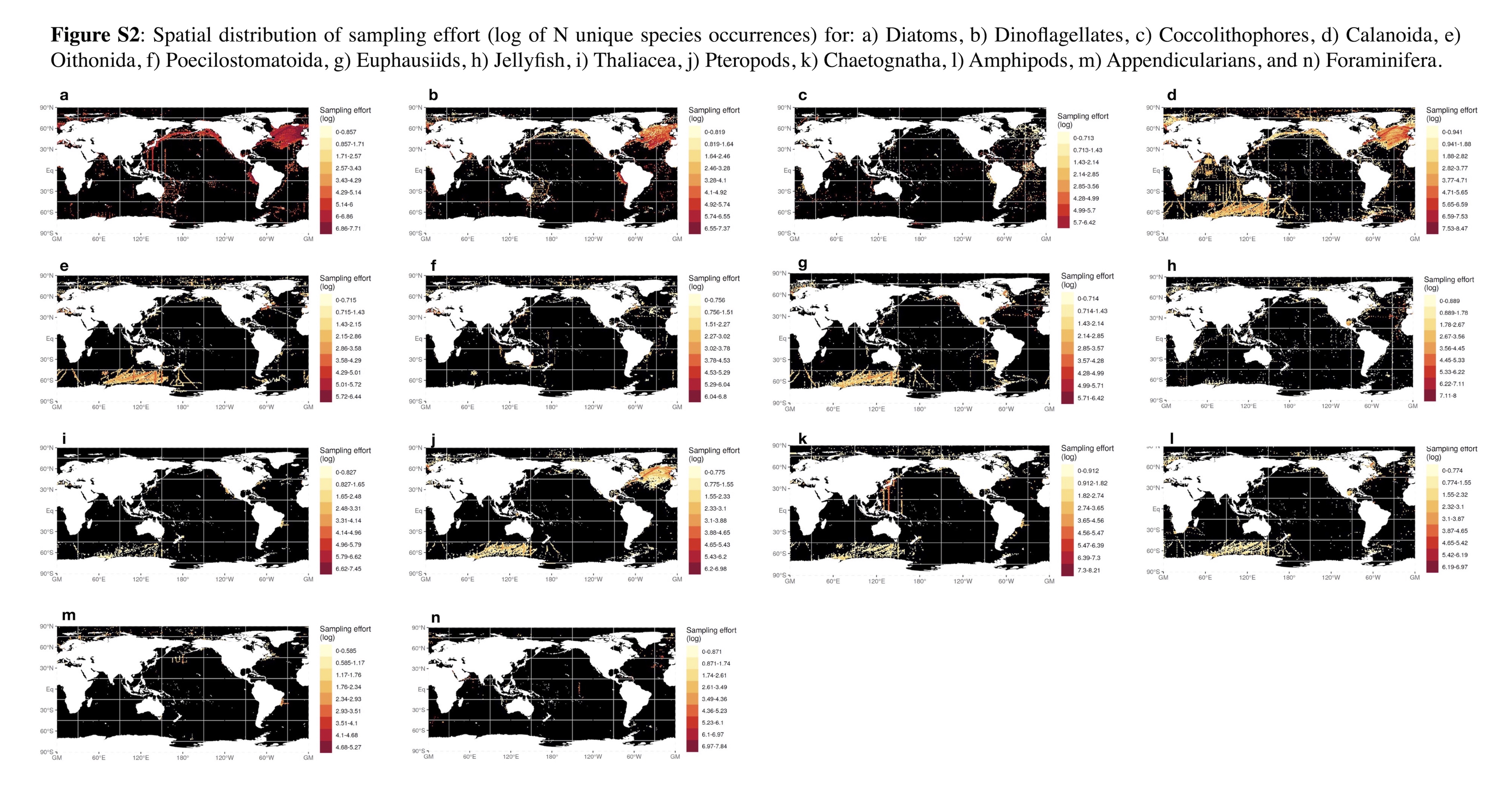

Supplement: Figure_S2_fbad044 [file figure_s2_fbad044.jpeg]

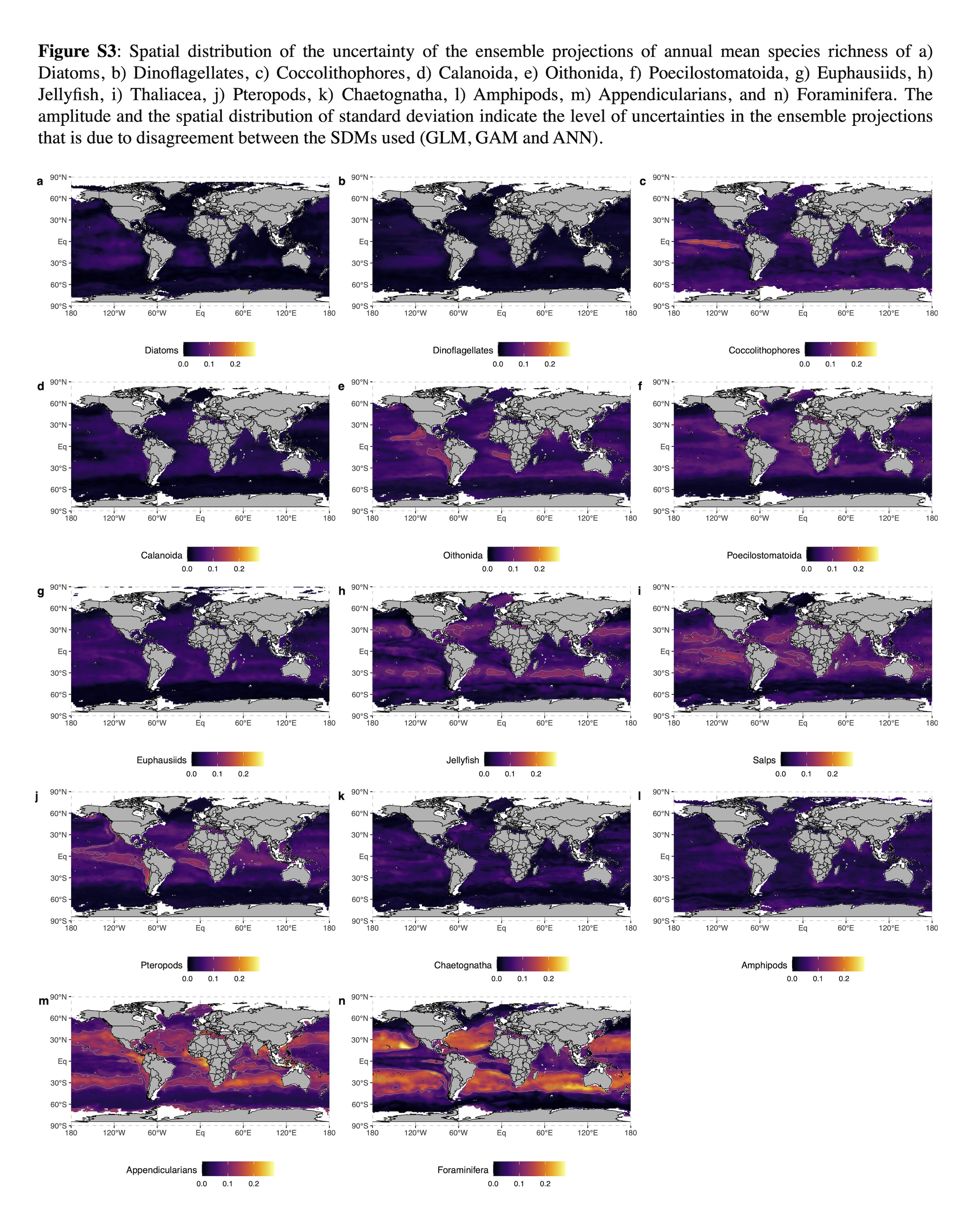

Supplement: Figure_S3_fbad044 [file figure_s3_fbad044.jpeg]

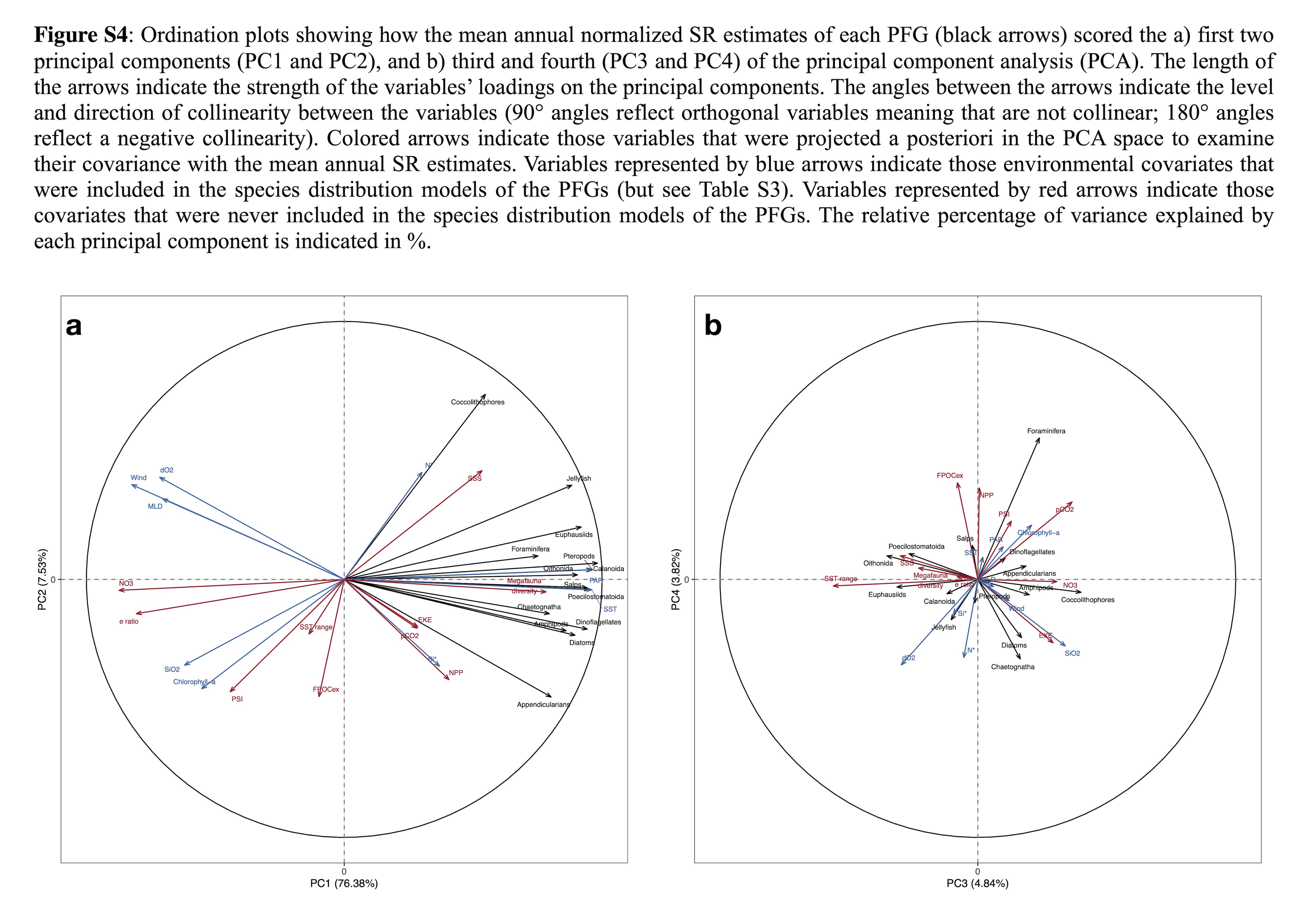

Supplement: Figure_S4_fbad044 [file figure_s4_fbad044.jpeg]
